# High Prevalence of Macrolide-resistance and Molecular Characterization of *Streptococcus pyogenes* Isolates Circulating in China from 2009 to 2016

**DOI:** 10.3389/fmicb.2017.01052

**Published:** 2017-06-08

**Authors:** Binghuai Lu, Yujie Fang, Yanyan Fan, Xingchun Chen, Junrui Wang, Ji Zeng, Yi Li, Zhijun Zhang, Lei Huang, Hongxia Li, Dong Li, Fengxia Zhu, Yanchao Cui, Duochun Wang

**Affiliations:** ^1^Department of Laboratory Medicine, Civil Aviation General Hospital, Peking University Civil Aviation School of Clinical MedicineBeijing, China; ^2^State Key Laboratory for Infectious Disease Prevention and Control, Chinese Centre for Disease Control and Prevention, National Institute for Communicable Disease Control and PreventionBeijing, China; ^3^Collaborative Innovation Centre for Diagnosis and Treatment of Infectious DiseasesHangzhou, China; ^4^Laboratory of Clinical Microbiology and Infectious Diseases, Department of Pulmonary and Critical Care Medicine, China-Japan Friendship HospitalBeijing, China; ^5^Department of Laboratory Medicine, People's Hospital of Guangxi Zhuang Autonomous RegionNanning, China; ^6^Department of Clinical Laboratory, Affiliated Hospital of Inner Mongolia Medical UniversityHohhot, China; ^7^Department of Laboratory Medicine, Wuhan Pu Ai Hospital of Huazhong, University of Science and TechnologyWuhan, China; ^8^Department of Laboratory Medicine, Henan Provincial People's HospitalZhengzhou, China; ^9^Department of Laboratory Medicine, Tai'an City Central Hospital (Tai'an)Shandong, China; ^10^Department of Laboratory Medicine, First Hospital, Peking UniversityBeijing, China; ^11^Department of Laboratory Medicine, Chengdu First People's Hospital (Chengdu)Sichuan, China

**Keywords:** molecular characterization, antibiotic resistance, *Streptococcus pyogenes*

## Abstract

*Streptococcus pyogenes*, or group A *Streptococcus*, is a pathogen responsible for a wide range of clinical manifestations, from mild skin and soft tissue infections and pharyngitis to severe diseases. Its epidemiological characteristics should be comprehensively under surveillance for regulating the national prevention and treatment practice. Herein, a total of 140 *S. pyogenes*, including 38 invasive and 102 noninvasive isolates, were collected from infected patients in 10 tertiary general hospitals from 7 cities/provinces in China during the years 2009–2016. All strains were characterized by classical and molecular techniques for its *emm* types/subtypes, virulent factors and antibiotic resistance profiling. Of 140 isolates, 15 distinct *emm* types and 31 subtypes were detected, dominated by *emm*12 (60 isolates, 42.9%), *emm*1(43, 30.7%), and *emm*89 (10, 7.1%), and 8 new *emm* variant subtypes were identified. All strains, invasive or not, harbored the superantigenic genes, *speB* and *slo*. The other virulence genes, *smeZ, speF*, and *speC* accounted for 96.4, 91.4, and 87.1% of collected isolates, respectively. Further multilocus sequence typing (MLST) placed all strains into 22 individual sequence types (STs), including 4 newly-identified STs (11, 7.9%). All isolates were phenotypically susceptible to penicillin, ampicillin, cefotaxime, and vancomycin, whereas 131(93.5%), 132(94.2%), and 121(86.4%) were resistant to erythromycin, clindamycin, and tetracycline, respectively. Our study highlights high genotypic diversity and high prevalence of macrolide resistance of *S. pyogenes* among clinical isolates circulating in China.

## Introduction

*Streptococcus pyogenes*, or Lancefield group A streptococcus, is an essential cause of morbidity and mortality worldwide nowadays, leading to a wide range of human infections, ranging from mild to life-threatening invasive infections (Koutouzi et al., 2015; Plainvert et al., 2016).

A variety of virulence factors contribute to pathogenesis. The M protein, encoded by *emm* gene, is a surface protein and virulence factor of *S. pyogenes*, capable of adhering to the epithelium and conferring protection against macrophage killing (Sanderson-Smith et al., 2014). Furthermore, it harbors a highly variable region with the antigen, and the protective role of M-protein type-specific antibody has been confirmed (Baroux et al., 2014; Sanderson-Smith et al., 2014). Moreover, *emm* gene sequencing is a standard method for typing M protein, and the distribution of *emm* types demonstrated a high degree of variability in line with geographic location, time, and types of clinical infections (Steer Ac et al., 2009). Therefore, identification of *emm* type is extremely useful for understanding the local epidemiological characteristics. Moreover, M-type specific antibodies are only responsible for immunity against the homologous M-type, with no effect on infection by heterologous M-types (Sanderson-Smith et al., 2014); therefore, continuous surveillance of *emm* types is consequently needed to evaluate the possible benefits of an M protein-based *S. pyogenes* vaccine (Steer Ac et al., 2009). Recently, a new *emm*-cluster typing system that classifies the many *S. pyogenes emm* types into 48 discrete *emm* clusters containing closely related M proteins that share binding and structural properties has been proposed (Sanderson-Smith et al., 2014). This system might predict the M protein vaccine antigen content and serve as a framework to investigate the cross-protection phenomenon between different *emm*-types (Baroux et al., 2014; Sanderson-Smith et al., 2014).

Besides, streptococcal pyrogenic exotoxins (*spe*) also play a crucial role in the pathogenesis of *S. pyogenes* infections by acting as superantigens (*spe*A-C and *spe*F-M). Moreover, streptococcal superantigen A (*ssa*), streptococcal mitogenic exotoxin Z (*sme*Z), and other virulence proteins, including C5a peptidase (*scp*A), streptokinase (*ska*), streptolysin O (*slo*), streptolysin S (*sag*A), and extracellular phospholipase A (*sla*), also constitute the main epidemiological characteristics of the local *S. pyogenes* isolates.

Multilocus sequence typing (MLST) has been developed for identifying clones of *S. pyogenes* isolates as epidemic feature (Plainvert et al., 2016). Finally, previous documents have reported the increased resistance of *S. pyogenes* strains to erythromycin (Bingen et al., 2004), clindamycin (Bingen et al., 2004), and fluoroquinolone (Lin et al., 2015).

The distribution of the above-mentioned characteristics constitutes the main epidemiology of local *S. pyogenes* isolates. The changing epidemic characteristics should be under continuous surveillance for effective prevention and treatment (Bingen et al., 2004; Kim et al., 2013; Wajima et al., 2013; Seale et al., 2016). However, scarce data on *S. pyogenes* infections are available in mainland China, except for a few studies conducted locally with isolates mainly from children with scarlet fever (Liang et al., 2012; Yang et al., 2013). Therefore, the aim of our work is to elucidate the characterization of serotypes, potential virulence factors, evolutionary relationship, and antibiotic resistance of *S. pyogenes* strains circulating in China. Furthermore, comprehensive genetic relationships deduced from different molecular types will be illustrated.

## Materials and methods

### Ethical statement

This study was exempted from review by the ethics committee of Civil Aviation General Hospital (CAGH), Beijing, because it focused on the epidemical characteristics of *S. pyogenes*.

### Strain collection and identification

A total of nonredundant 140 *S. pyogenes* isolates responsible for human infection were included in current study. The patients enrolled were aged from 2 months to 92 years, with the median age at 19.6 years; 47.1% were female. The isolates were obtained from bloodstream (16 cases), surgical-site, skin/soft tissue infection, and other wound secretions (wound, 18; skin/soft tissue infection, 7), abscess (16), pharyngitis, and/or tonsillitis (pharyngeal swab, 52), respiratory tract (bronchial alveolar lavage fluid, BALF, 3, and phlegm, 10), reproductive tract infection (abscess of vulva, 6), biopsy tissue (1), ear pus (5), pleural effusion (4), urine (1), and pyoderma gangraenosum (1) of patients who visited CAGH (Beijing) during 2009–2016, Peking University First Hospital (Beijing) during 2010–2014, Affiliated hospital of Inner Mongolia medical university (Huhehot, Inner Mongolia Autonomous region) during 2011–2014, Henan Provincial People's Hospital (Zhengzhou, Henan Province) during the period 2014–2015, Wuhan Pu Ai Hospital of Huazhong University of Science and Technology (Wuhan, Hubei Province) during 2013–2015, People's Hospital of Guangxi Zhuang Autonomous Region (Nanning, Guangxi Zhuang Autonomous Region) during 2009–2016, Beijing Tsinghua Chang Gung Hospital, Medical Center of Tsinghua University (Beijing) during 2014–2016, Beijing Tongren Hospital (Beijing) during 2012, Tai'an City Central Hospital (Tai'an, Shandong Province) during the period of 2014–2016, and Mianyan Central Hospital (Mianyang, Sichuan Province) during the period of 2015. The isolates were sent to the Department of Clinical microbiology of CAGH for further confirmation by β-hemolysis on sheep blood agar, grouping of carbohydrate antigen (Streptococcal grouping kit, Oxoid), and 16S rRNA gene sequencing using the primers BAK11w and BAK2, as described previously (Lu et al., 2013).

### Case definition

Definite invasive disease was defined by the isolation of *S. pyogenes* from a normally-sterile site (e.g., blood, cerebrospinal, pleural, or peritoneal fluid). Noninvasive infection was defined as isolation of the microorganism from a nonsterile site with a clinical syndrome consistent with S. *pyogenes* infection but that did not meet the probable invasive *S. pyogenes* disease case definition (Friaes et al., 2013; Baroux et al., 2014).

### *Emm* typing

All *S. pyogenes* isolates were subjected to *emm* typing. The *emm* gene was amplified by PCR (http://www.cdc.gov/streplab/protocol-emm-type.html), DNA fragments were sequenced and aligned by the comparison of the query sequence with the reference database (ftp://ftp.cdc.gov/pub/infectious_diseases/biotech/tsemm/, Centers for Disease Control and Prevention, Atlanta, GA, USA). The newly-identified sequences defining new *emm* subtypes were re-sequenced in order to validate the initial findings, then submitted, and assigned to a new subtype. The assignment of *emm* clusters was based on the CDC database (http://www.cdc.gov/streplab/downloads/distribution-emm-types.pdf) as previously described (Baroux et al., 2014).

### MLST

MLST was performed by sequencing seven housekeeping genes (*gki, gtr, murI, mutS, recP, xpt*, and *yqiL*; Enright et al., 2001; Wu et al., 2008). After each gene was amplified by PCR, DNA fragments were sequenced. Then, the sequence types (STs) and allelic profiles were performed using MLST database (http://spyogenes.mlst.net/). Each isolate was assigned to an ST. An ST not identified in any cluster was assigned as a singleton. BioNumerics software version 5.1 (Applied Maths, Belgium) was used to create minimum spanning trees to illustrate the relationships between MLST and *emm* typing. The newly identified alleles and those alleles defining new STs were submitted to the MLST database curator for approval and a number was assigned. The isolates were assigned to one of the clonal complexes (CC) if they shared four or more alleles with the predominant ST.

### Virulence genes

Virulence genes were detected by using conventional PCR amplification method. The target genes included *S. pyogenes* exotoxin and Streptococcal superantigenic genes (*spe*A–C, s*pe*F–M, *ssa, and sme*Z), and other specific virulence genes (*sil, slo, and sla*), using primer pairs described previously (Friães et al., 2013; Lu et al., 2016).

### Antibiotic susceptibility testing and antimicrobial resistance genes

The broth microdilution method was used to determine the susceptibility of all isolates to penicillin G (range of concentration tested: 0.03–4 μg/mL), ampicillin (0.03–4 μg/mL), cefotaxime (0.03–4 μg/mL), erythromycin (0.03–4 μg/mL), clindamycin (0.03–4 μg/mL), levofloxacin (0.25–32 μg/mL), tetracycline (0.255–32 μg/mL), and vancomycin (0.03–4 μg/mL). The cation-adjusted Mueller-Hinton broth with lysed horse blood (2.5–5% v/v) was provided by Tianjing Jinzhang Science and Technology Development, China. The results were interpreted in accordance with the breakpoints set for *Streptococcus spp*. β-hemolytic group by the Clinical and Laboratory Standards Institute (CLSI, 2016). The macrolide-resistant isolates were further classified as having the cMLSB (constitutive macrolide-lincosamide-streptogramin B resistance), iMLSB (inducible resistance), or M phenotype (macrolide-streptogramin B resistance and lincosamide susceptibility) by the double-disc synergy test.

Genetic determinants of *erm* (A) (subclass *erm*TR), *erm* (B), and *mef* (A/E) were investigated by PCR in erythromycin-resistant isolates as we described previously (Lu et al., 2016). Furthermore, the resistance genes of tetracycline, including *tet* (M), *tet* (K), *tet* (L), and *tet* (O), were determined in tetracycline-resistant isolates using PCR as described elsewhere (Lu et al., 2016). PCR products were selected for sequencing and aligned by BLAST software to confirm the correctness. The genes coding DNA gyrase A (*gyrA*) and topoisomerase IV C (*parC*) were amplified, sequenced and aligned in all isolates, to determine fluoroquinolone resistance and the sequence substitutions by using the primers previously proposed (Lu et al., 2016). Comparison analyses of sequences were conducted with BioEdit software (Ibis Biosciences, Carlsbad, CA, USA). Clustal-W was used to perform multiple alignments of the nucleotide and predicted amino acid sequences. The reference sequences of *gyr*A (*gyr*A-ATCC 700294, AF220945.1) and *par*C (*par*C-ATCC 700294, AF220946.1) were accessed from GenBank.

### Statistical method

Chi square (χ^2^)-test was used to analyze different infection types by year and *emm* type using SPSS 17.0 software, and significance was defined when the *P*-value was < 0.05.

## Results

### Distribution of sources of *S. pyogenes* isolates and *emm* distribution

A total of 140 *S. pyogenes* isolates collected from 10 tertiary hospitals in China were enrolled in the study, including 38(27.1%) of invasive and 102(72.9%) of noninvasive. The isolates were recovered from 66 women and 74 men (ratio 1:1), and 63 (45%) from children (≤14 years). Compared by year, the infection caused by invasive and noninvasive infection was different in different years (χ^2^ = 19.150, *p* < 0.008). Details of *S. pyogenes* isolates recovered are shown in Table 1.

**Table 1:** Demographic characteristics of patients infected with *S. pyogenes* during 2009–2016 in China.

**Year**	**No. strains (n = 140)**	**Infection type**		**Gender**		Cities/Provinces (n = 140)^*^	Sample resources (n = 140)^*^
		**Invasive (38)**	**Noninvasive (102)**	**Male (74)**	**Female (66)**		
2009	9	4	5	5	4	Beijing (2), Henan(6), Hunan	Bloodstream(4), wound(3), phlegm, urine
2010	3	3	0	2	1	Beijing(2), Hunan	Abscess(3)
2011	4	3	1	2	2	Beijing, Henan(2), Inner mongolia	Abscess(2), bloodstream, wound
2012	10	3	7	5	5	Beijing(6), Hunan, Inner mongolia(3)	Abscess(3), wound(3), phlegm(2), BALF, pharyngeal swab
2013	3	1	2	2	1	Beijing, Hunan, Inner mongolia	Bloodstream, wound, pharyngeal swab
2014	11	5	6	5	6	Beijing, Inner mongolia(10),	Wound(4), bloodstream(3), abscess(2), pharyngeal swab, phlegm
2015	51	8	43	32	19	Beijing(20), Guangxi(11), Inner mongolia(4), Sichuan(16)	Abscess(2), abscess of vulva, BALF, bloodstream(3), ear pus(3), pharyngeal swab(26), phlegm(4), pleural effusion(2), pyoderma gangraenosum, skin/soft tissue infection(5), wound(3)
2016	49	11	38	21	28	Beijing(30), Guangxi(12), Hunan(3), Shandong(4),	Abscess(4), abscess of vulva(5), BALF, biopsy tissue, bloodstream(4), ear pus(2), pharyngeal swab(23), phlegm(2), pleural effusion(2), skin/soft tissue infection(2), wound(3)

* *Number in parentheses represents strains, and no number signified only one strain was detected; BALF, bronchial alveolar lavage fluid*.

**Figure 1:**
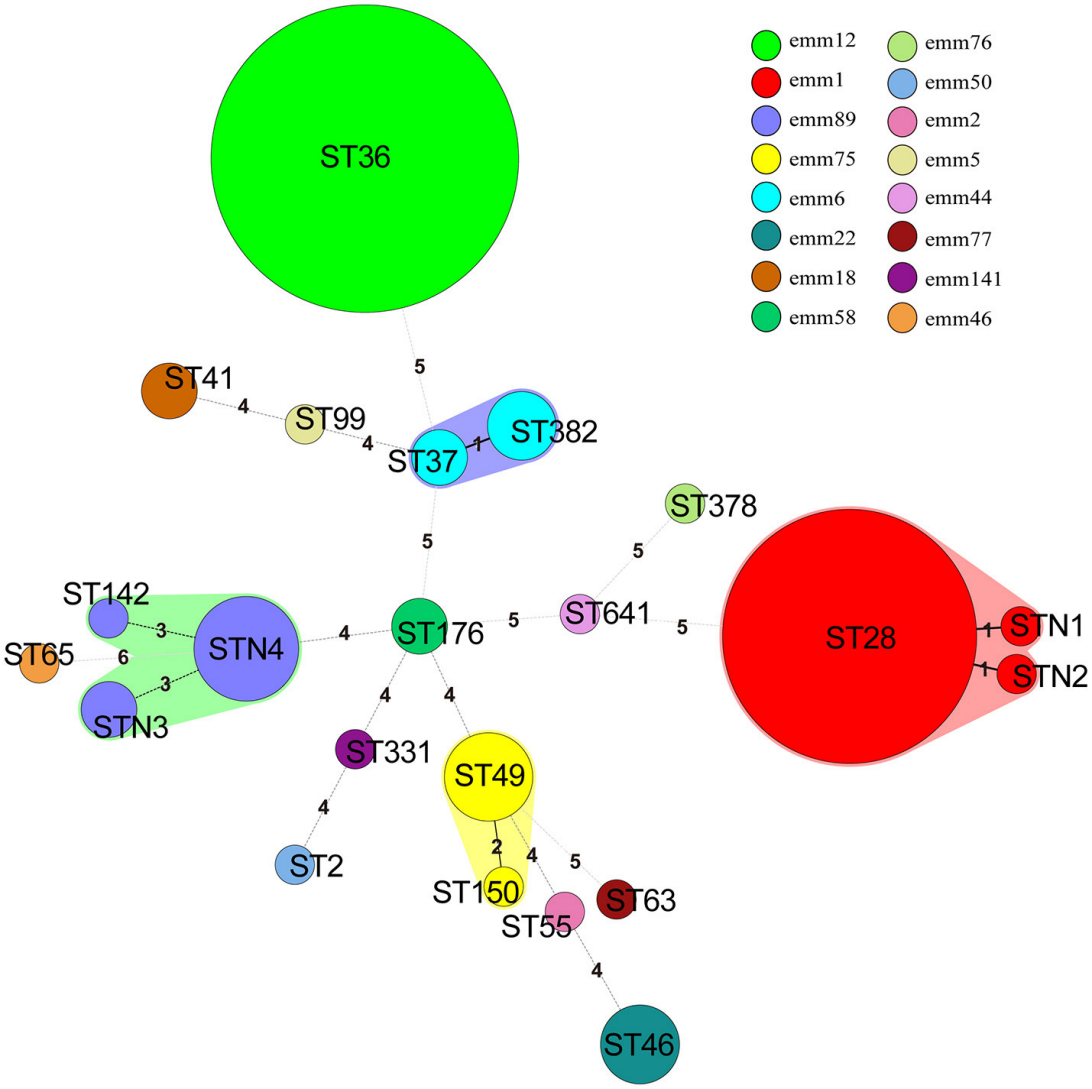
Minimum spanning tree of 140 *S. pyogenes* isolates in China based on Multilocus sequence typing (MLST). The size of each circle indicates the number of isolates within this particular type. The colors of the halo surrounding the STs denote types that belong to the same clonal complex. *Emm* types are represented by different colors. The number between different circles represented the number of locus variants.

The *emm* type distribution for 140 *S. pyogenes* strains is demonstrated in Table 2. A total of 15 distinct *emm* types and 31 subtypes were identified. Overall, the prevalent *emm* types were *emm*12 (60, 42.9%), *emm*1 (43, 30.7%), *emm*89(10, 7.1%), and *emm*75 (6, 4.3%), accounting for 85.0% of the total population, as shown in Figure 1, Table 2. However, there was no difference between invasive and noninvasive infections caused by different *emm* types (χ^2^ = 23.566, *p* < 0.073). The *emm* types in current study belonged to 8 *emm* clusters. Among them, there were 3 most prevalent clusters, A-C3, A-C4, and E4, accounting for 43 (30.7%), 61 (43.6%), and 15(10.7%), respectively, comprising 85.0% of all isolates. For the 16 isolates causing bloodstream infection, the predominated *emm* types were *emm*12 (4 isolates) and 1 (4). In addition, 8 new *emm* types/subtypes were newly-identified in current study (*emm*1.85, *emm*12.40, *emm*12.93, *emm*12.94, *emm*18.43, *emm*46.3, *emm*5.141, and *emm*141.1), those *emm* sequences were submitted to GenBank under accession number KY697798-KY697805.

**Table 2:** Distribution of *emm* types, *emm* cluster, and sequence types of *S. pyogenes* isolated during 2009–2016 in China.

***emm* typing**	*emm* subtypes (n)^*^	***emm* clustuers**	ST (n)^*^	**No. strains (n = 140)**	**Percent (%)**
*emm*12	*emm*12.0(44), *emm*12.19(9), *emm*12.72(3), *emm*12.40 (2), *emm*12.93, *emm*12.94	A-C4	ST36(60)	60	42.9
*emm*1	*emm*1.0(35), *emm*1.25, *emm*1.33(2), *emm*1.40, *emm*1.76, *emm*1.85 (3)	A-C3	ST28(41),STN1,STN2	43	30.7
*emm*89	*emm*89.0(10)	E4	ST142,STN3(2),STN4(7)	10	7.1
*emm*75	*emm*76.0(6)	E6	ST49(5),ST150	6	4.3
*emm*6	*emm*6.4(3), *emm*6.7(2)	SPECY	ST382(3), ST37(2)	5	3.6
*emm*22	*emm*22.0(3), *emm*22.1	E4	ST46(4)	4	2.9
*emm*58	*emm*58.0, *emm*58.7	E3	ST176(2)	2	1.4
*emm*18	*emm*18.0, *emm*18.43	SPECY	ST41(2)	2	1.4
*emm*2	*emm*2	A-C4	ST55	1	0.7
*emm*50	*emm*50	E2	ST2	1	0.7
*emm*76	*emm*76	E2	ST378	1	0.7
*emm*44	*emm*44	E3	ST641	1	0.7
*emm*46	*emm*46.3	A-C1	ST65	1	0.7
*emm*77	*emm*77	E4	ST63	1	0.7
*emm*5	*emm*5.141	SPECY	ST99	1	0.7
*emm*141	*emm*141.1	TBD	ST331	1	0.7

* *Number in parentheses represents strains, and no number signified only one strain was detected. TBD, to be determined; ST, sequence typing; SPECY, Single protein emm-cluster clade Y*.

### Distribution of virulence-associated genes

**Figure 2:**
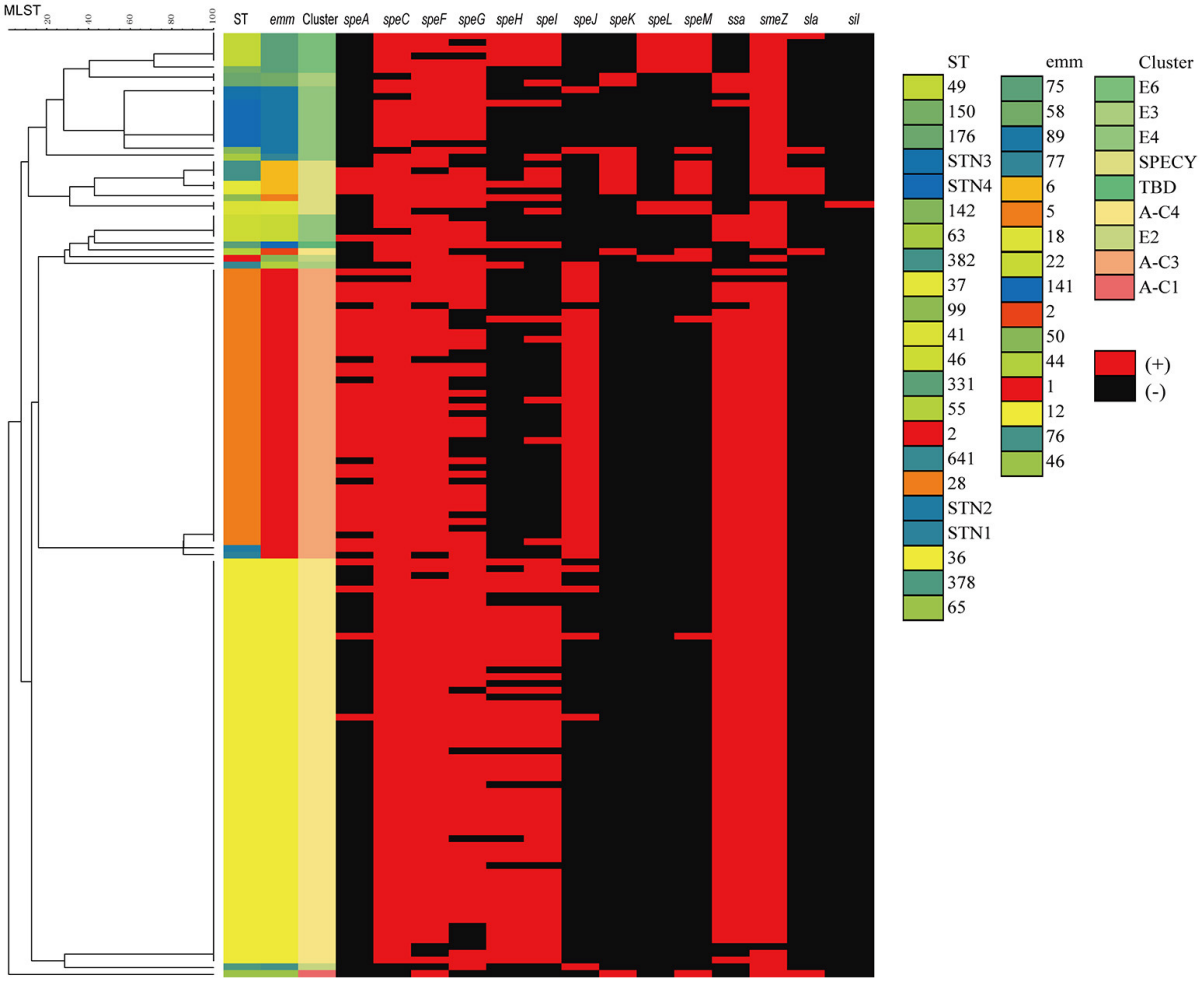
Dendrogram constructed from the multilocus sequence typing (MLST) profiles of seven housekeeping genes of 140 *S. pyogenes* isolates circulating in 10 hospital settings in China, showing the distributions of virulence genes, *emm* type and MLST. SPECY, Single protein *emm*-cluster clade Y. TBD, to be determined.

A total of 16 virulence genes were identified by PCR methods. All strains, invasive or not, harbored the 2 superantigenic toxic genes: *spe*B and *slo*. Of other virulence genes, *sme*Z, *spe*F and *spe*C were prevalent, accounting for 96.4% (135/140), 91.4% (128/140), and 87.1% (122/140), of collected isolates, respectively. By comparison, the percentage of isolates that harbored *sil* (1.4%, 2/140), *sla* (5.7%, 8/140), *spe*L (6.4%, 9/140), and *speK* (7.9%, 11/140) was extremely low. As demonstrated in Figure 2, the close relationship was notable between *emm* type and virulence genes, for example, only 2 (1.4%) *emm*12 *S. pyogenes* isolates contained *speJ*, while most *emm*1(42, 97.7%) harbored it; Only 1 (0.7%) and 5 (3.6%) *emm*1 *S. pyogenes* isolates contained *speH* and *speI*, but most *emm*12 harbored them, accounting for 45(75.0%) and 46 (76.7%) isolates, respectively.

### MLST and relationship with *emm* typing

All 140 isolates were selected for MLST analysis, and a total of 22 individual STs were distinguished (Table 2, Figure 1), including 4 new STs (*n* = 11, 7.9%) designated as STN1, STN2, STN3, and STN4, respectively. In the alignment of MLST sequence, 3 novel sequences were found, *xpt* in isolate KT500, *recP* in KT523, and *gtr* in both KT205 and KT525, which were designated as *xpt*1, *recP*1, and *gtr*1, respectively. Moreover, 92.1% (129/140) isolates were represented by 10 main STs (having ≧2 isolates), and the two most prevalent STs were ST36 and ST28, accounting for 42.9% (60/140) and 29.3% (41/140), respectively. Besides, MLST analysis indicated that each *emm* type was almost corresponding to a single ST, or at least, a clonal complex, e.g., *emm*1 and ST28, *emm*12, and ST36, as shown in Table 2, Figure 1.

### Phenotype and genetic profile of antimicrobial susceptibility

All isolates collected in current study were phenotypically susceptible to penicillin, ampicillin, cefotaxime, and vancomycin.

Of 140 *S. pyogenes* isolates, 131 were resistant, 2 intermediate, and 7 susceptible, to macrolide; 132 *S. pyogenes* isolates were resistant, 1 intermediate, and 7 susceptible, to clindamycin. No M phenotype was detected. Of all erythromycin-resistant cMLSB isolates, cMLSB/*erm* (B) phenotype/genotype was the most frequently identified (116, 82.9%), while the combination of *erm* (B)+*mef* (A/E) and *erm* (B)+*erm* (A) were detected only in 6 and 4 isolates. The 5 isolates of iMLSB phenotype harbored exclusively *erm* (B) gene. Interestingly, all the *S. pyogenes* isolates in current study, phenotypically susceptible or resistant to macrolide, harbored *erm* (B) gene.

Of 140 *S. pyogenes* isolates in current study, 17 were susceptible, 2 intermediate, and 121 resistant to tetracycline. In 121 resistant isolates, no *S. pyogenes* isolate carried *tet* (K) gene, only one isolate harbored *tet* (L) combined with *tet* (M), and only 9 tetracycline-resistant isolates carried *tet* (O), singly or combined; comparatively, most isolates (116/121, 95.9%) carried *tet* (M). Notably, 1 out of the 2 intermediate isolates, and 7 out of 17 susceptible were also positive for *tet* (M), as shown in Table 3.

**Table 3:** Association of erythromycin and tetracycline phenotype and genotype and the distribution of *emm* and sequence typing; in 140 *Streptococcus pyogenes* isolated during 2009–2016 in China.

**Phenotype (n)**	**Genotype**	No. of strains (%)^*^	**ST (n)^#^**	***emm* cluster^#^**
**ERYTHROMYCIN**				
cMLSB(126)	*erm*(B)	116(82.9)	STN1,STN2,STN4(6),ST46(3),ST49(5),ST65,ST150, ST378,ST382,ST641,ST36(56),ST41,ST28(38)	A-C1,A-C3(40),A-C4(56),SPECY(2),E2,E3,E4(9),E6(6)
	*erm*(B) *+erm*(A)	6(4.3)	ST28,ST41,ST63,ST176,STN3(2)	A-C3, SPECY, E3,E4(3)
	*erm*(B) *+mef*(A/E)	4(2.9)	ST176,ST382(2),ST331	E3,TBD,SPECY(2)
iMLSB(5)	*erm*(B)	5(3.6)	ST36(3),ST46,ST99	A-C4(3), SPECY, E4
L Phenotype(1)	*erm*(B)	1(0.7)	STN4	E4
S(6)	*erm*(B)	6 (4.3)	ST2,ST28(2),ST37,ST55,ST142	E2,A-C3(2),SPECY, E4(2)
I(2)	*erm*(B)	2(1.4)	ST36,ST37	A-C4, SPECY
**TETRACYCLINE**				
S(17)	*tet*(M)	7(5)	ST28,ST3(3),ST46,ST49,STN4	A-C3, A-C4(3), E4(2), E6
	*None*	10(7.1)	ST28,ST37,ST46,ST49(4),ST55,ST150,ST331	A-C3, SPECY, E6(5), E4(2), TBD
I(2)	*None*	1(0.7)	ST28	A-C3
	*tet*(M)	1(0.7)	ST382	SPECY
R(121)	*tet*(M)	108(77.1)	ST28(31),ST36(55),ST37,ST41,ST46(2),ST65,ST99,ST142, ST176(2),ST378,ST382,ST641,STN1,STN2,STN3(2),STN4(6)	A-C1, A-C3(33), A-C4(55), SPECY(4), E2, E3(3),E4(11)
	*tet*(M)+ *tet*(L)	1(0.7)	ST28	A-C3
	*tet*(M)+ *tet*(O)	7(5)	ST28(3),ST36(2),ST41,ST382	A-C3(3), A-C4(2), SPECY(2)
	*tet*(O)	2(1.4&)	ST2,ST63	E2,E4
	*None*	3(2.1)	ST28(3)	A-C3(3)

* Percentage in parentheses represents isolates/total isolates (140);

# *Number in parentheses represents strains, and no number signified only one strain was detected. cMLSB, constitutive macrolide-lincosamide-streptogramin B resistance; iMLSB, inducible macrolide-lincosamide-streptogramin B resistance; L, L Phenotype, macrolide-streptogramin B susceptibility and lincosamide resistance; S/I/R: the isolates susceptible/intermediate/resistant to macrolide or tetracycline; ST: sequence typing;TBD: to be determined; SPECY, Single protein emm-cluster clade Y*.

### Mutation of *gyrA* and *parC* leading to resistance to fluoroquinolone

Fluoroquinolone resistance-related genes, *gyrA*, and par*C*, were amplified and sequenced. Regarding to *S. pyogenes*, the gene *gyrA* was highly conserved, exhibiting limited variation, and nonsynonymous mutations were only observed in 3 isolates, M99L for isolate KT528, S81F for KT191, and V192I for PCR75 with an MIC at 0.75, >256, and >256 μg/mL, respectively. By comparison, multiple amino acid point mutations were identified in the *parC* sequences (61.4%, 86/140), both in susceptible and resistant isolates. Among 140 *S. pyogenes* isolates, 5 were non-susceptible isolates, with details in Table 4. They were obviously sporadically-distributed in various *emm*-types and infection sites. Moreover, in 135 fluoroquinolone-susceptible *S. pyogenes* isolates in current study, A121V (59, 43.7%) and D91N (16,11.4%) were the most prevalent mutations. Meanwhile, the close relationship between *parC* mutation and *emm* types was notable, for example, all *emm*12 strains, resistant or not, contained A121V, however, almost no fluoroquinolone-susceptible *emm*1 strains harbored mutation (except 1 isolate having a mutation of S79-F), *emm*6 harbored S79A mutation, and *emm*89 harbored both D91N and S140P mutations, as shown in Table 5.

**Table 4:** Phenotype, genotype, distribution of *emm*, and other characteristics of 5 fluoroquinolone non-susceptible *Streptococcus pyogenes* isolates in China.

**No**.	**Erythromycin phenotype and genotype**	***emm* typing**	***emm* cluster typing**	**Infection types**	**ST**	**MIC(μg/mL)**	***gyrA***	***parC***
KT405	cMLSB/*erm*(A)+*erm*(B)	*emm*12	A-C4	abscess	ST36	3/I	–	S79F,A121V
KT675	cMLSB/*erm*(B)	*emm*89	E4	skin/soft tissue infection	STN4	3/I	–	D91N,S140P
PCR75	cMLSB/*erm*(B)	*emm*44	E3	wound	ST641	>256/R	V192I	–
PCR76	cMLSB/*erm*(B)	*emm*12	A-C4	wound	ST36	>256/R	–	A121V
KT191	cMLSB/*erm*(A)+*erm*(B)	*emm*58	E3	abscess	ST176	>256/R	S81F	S79F

*ST, sequence typing; cMLSB, constitutive macrolide-lincosamide-streptogramin B resistance*.

**Table 5:** Characteristics of amino acid substitutions of *ParC* in 135 fluoroquinolone(Levofloxacin)-susceptible *Streptococcus pyogenes* isolates in China.

**Point mutation in *ParC***	No.(%)^*^	**ST^#^**	***emm*-cluster typing^#^**	***emm* typing^#^**
A121V	55(40.7)	ST36(54),ST378	A-C4(54),E2	*emm*12(54), *emm*76
S79Y and A121V	3(2.2)	ST36(3)	A-C4(3)	*emm*12(3)
D83V and A121V	1(0.7)	ST36	A-C4	*emm*12(36)
D91N	3(2.2)	ST46(3)	E4(3)	*emm*22(3)
D91N and S140P	10(7.4)	ST64,142, STN3(2), STN4(6)	E4(10)	*emm*77, *emm*89(9)
S140P	2(1.5)	ST41(2)	SPECY(2)	*emm*18(2)
S79F	2(1.5)	ST2,ST28	A-C3,E2	*emm*1, *emm*50
S79F and D91N	2(1.5)	ST46,ST150	E4,E6	*emm*22, *emm*75
S79A	5(3.7)	ST37(2), ST382(3)	SPECY(5)	*emm*6(5)
*None*	52(38.5)	ST28(40),STN1,STN2,ST55,ST65,ST176,ST49(5),ST331,ST99	A-C3(42), A-C4, A-C1,E3, E6(5),SPECY,TBD	*emm*1(42), *emm*46, *emm*58, *emm*75(5), *emm*5, *emm*141

* Percentage in parentheses represents isolates/total fluoroquinolone(Levofloxacin)-susceptible isolates (135);

# *Number in parentheses represents strains, and no number signified only one strain was detected. SPECY, Single protein emm-cluster clade Y. TBD: to be determined*.

## Discussion

*S. pyogenes* is an important pathogen involved in a wide variety of human infections and a major cause of morbidity and mortality worldwide, ranging from noninvasive diseases, such as acute pharyngitis, to life-threatening invasive infections, such as sepsis and toxic shock syndrome (Gherardi et al., 2015). Herein, we characterized a collection of 140 *S. pyogenes* isolates in China during 2009–2016 and compared them with *S. pyogenes* isolates from around the world.

The distribution of *emm* genes of *S. pyogenes* strains should be under continuous surveillance, for its variety in different regions and changing with time make wide implementation of universal M protein vaccine difficult. In present study, 95.7% (134/140) isolates were covered by the 26-valent M-protein-based *S. pyogenes* vaccine currently demonstrating to be immunogenic and safe in human trials (Steer Ac et al., 2009; Tamayo et al., 2014). Based on above epidemiological features, this vaccine will have extensive coverage of *S. pyogenes* isolates in China. According to a review regarding to *S. pyogenes* infection worldwide, the most common *emm* types were *emm*1 and *emm* 12, accounting for 18.3 and 11.1%, followed by *emm*28 (8.5%), *emm*3 (6.9%), and *emm*4 (6.9%) (Steer Ac et al., 2009). Similarly, in current study, the leading *emm* types were *emm*12 (42.1%), *emm*1 (30.7%), *emm*89 (7.1%), and *emm*75 (4.3%), accounting for 84.3% of the total population. Our data during a 8-year period is different from that of Greece during 2007–2013 in children [(35 *emm* types in 1282 strains, with the most prevalent *emm* types being *emm*1 (16.7%), *emm*12 (13.6%), *emm*77 (10.9%), *emm*6 (6.8%), and *emm*89 (6.6%)](Koutouzi et al., 2015), New Caledonia in 2012 [47 *emm* types among 318 *S. pyogenes* strains, the 5 most frequent *emm* types (*emm*76, *emm*95, *emm*25, *emm*1, and *emm*93) were responsible for only 51% of the cases] (Baroux et al., 2014), Finland during 2008–2013 [72 *emm* types in 1122 invasive isolates, of which *emm*28 (26%), *emm*89 (12%), and *emm*1 (12%) were 3 most types)] (Smit et al., 2015), and Portugal during 2006–2009 (the 4 most prevalent *emm* types, *emm*1, *emm*89, *emm*3, and 6, accounted for 60% of 191 isolates recovered from normally sterile sites; Friães et al., 2013). Furthermore, the distribution of *emm* types in current study was different from that in the previous study in children with scarlet fever in Beijing, China (647 *S. pyogenes* isolates, *emm*12, and 1 accounting for 76.4 and 17.1% respectively; Yang et al., 2013). Changes in the predominant circulating *emm* types within relatively short time periods in individual regions might be explained by increased populations moving. By comparison, all *S. pyogenes* isolates in present study were collected from 10 hospitals across China, half patients were adults with clinical confirmed infections, and the majority of them had underlying diseases. Therefore, our results might be more representative of characteristics of *S. pyogenes* from mainland China. Moreover, the *emm* types of invasive and noninvasive strains were detected to be insignificantly related, suggesting that no specific types of M proteins were responsible for the invasion of *S. pyogenes* (Yang et al., 2013).

MLST allowed the comparison of genetic profiles of isolates recovered from various geographic regions. In this study, new gene sequences were identified in *xpt, recP*, and *gtr* and 4 novel STs designated. Moreover, isolates within 15 *S. pyogenes emm* types shared identical or nearly identical STs, demonstrating concordance between the *emm* type and genetic relatedness. Each *emm* type was almost exclusively associated with ST, e.g., *emm*1 with ST28, and *emm*6 with ST382 (Lin et al., 2015; Plainvert et al., 2016). The horizontal transfer of the *emm* genes in *S. pyogenes* seems to be less often.

Virulence factors have a critical role in the pathogencity of *S. pyogenes*. In current study, all strains, invasive or not, harbored *speB* and *slo*. The other virulence genes, *smeZ, speC*, and *speF*, were determined in over 90% isolates. By comparison, the percentage of isolates that harbored *sil, sla, speL*, and *speK* was <10%, indicating their lose from most *S. pyogenes* lineages in China. The distribution of superantigens might be varied in accordance with geographic areas. For example, in France, the genes of *speA, speC, ssa*, and *smeZ* were detected in 59, 37, 13, and 92% of *S. pyogenes* in adult with meningitis from 2003 to 2013, respectively (Plainvert et al., 2016). In Australia, *speG* and *smeZ* were reported to be present in the majority (90 and 95%), and *speC* in half, of 107 *S. pyogenes* isolates, and comparable with our data, the frequency of *speL* (8%) and *speK* (9%) were also rare (Commons et al., 2008). However, in Portugal, the *speG* and *smeZ* were present in over 90% *S. pyogenes* isolates, while *speJ* was found in only 45% of 191 *S. pyogenes* isolates recovered from normally sterile sites during 2006–2009 (Friães et al., 2013). Furthermore, the distribution of superantigen in current study was different from that found in *S. pyogenes* isolates from a previous study concerning children with scarlet fever in Beijing, China (Yang et al., 2013), in which almost all *emm*1 strains had *speC*, and *ssa* was the main superantigen of *emm*1 and *emm*12 isolates. This might be explained by our isolates having a wide coverage nationwide. Nevertheless, the above study demonstrated an extremely low number of *S. pyogenes* isolates harbored *speK, speL*, or *speM*, consistent to our data (Yang et al., 2013). This might comprise the main characteristics of *S. pyogenes* isolates in China. In conclusion, the distribution of superantigens varied dramatically, and the isolates with the identical *emm* type often shared a diverse profile.

Several antimicrobial drugs effectively treat *S. pyogenes* infections. Herein, all *S. pyogenes* isolates were susceptible to β-lactams and vancomycin, and the reduced susceptibility to penicillin and cephalosporin was not observed, suggesting that β-lactams were still the first-line antibiotics against *S. pyogenes*.

Moreover, macrolides have been recommended for patients allergic to β-lactams, and clindamycin is the preferred antibiotic in the treatment of patients with serious soft-tissue infections because of its ability to inhibit the production of several streptococcal virulence factors (Gherardi et al., 2015). Thus, it is critical to conduct continuous surveillance for macrolide- and clindamycin-resistance in *S. pyogenes*. In present study, approximately 90% *S. pyogenes* strains were resistant to erythromycin, clindamycin, and tetracycline, and similar resistance rates were also documented in two previous studies conducted in children with scarlet fever in Beijing, China (Liang et al., 2012; Yang et al., 2013). Previously, we have reported a high resistance rate in other two main β-hemolytic *Streptococcus, S. agalactiae*, and *S. dysgalactiae* subsp. *equisimilis* (SDSE), in China (Binghuai Lu et al., 2016; Lu et al., 2016). Put together, erythromycin and clindamycin might be unsuitable in treatment of infections due to β-hemolytic *streptococcus* in mainland China. However, in contrast, the extent of the resistance of *S. pyogenes* to the two antibiotics in many other countries has been very low. For example, in Finland, the *S. pyogenes* isolates collected from blood and skin/soft tissue showed an erythromycin and clindamycin resistance of 1.9 and 0.9% in 2008 and 8.7 and 9.2% in 2013, respectively, though a little increase (Smit et al., 2015). In addition, in current study, both *erm* and *mef (A/E)* genes were identified in erythromycin-resistant isolates, with *erm* (B) dominating. We observed a higher level of discordance between genotype and phenotype in *S. pyogenes*, and there were *erm* (B) genes in 9 erythromycin-susceptible isolates, as consistent with previous report in *S. agalactiae* (Dela Cruz et al., 2007) and SDSE isolates (Lu et al., 2016), hinting an alternate mechanism of resistance or a potential mutation in the 23S Rrna (Dela Cruz et al., 2007). Furthermore, 90.3% (421/466) harbored the *erm* (B) gene in *S. pyogenes* strains isolated from Beijing children (Liang et al., 2012). Put together, this might indicate that *erm* (B) be the predominant resistance genes in β-hemolytic *Streptococci*, in China (Binghuai Lu et al., 2016; Lu et al., 2016). *S. pyogenes* clones showing *emm* types strongly associated with erythromycin resistance may contribute to the overall prevalence of macrolide resistance. In Italy, the most prevalent macrolide resistance mediated by *mef* (A) was present in 92.2% of *emm*1 strains (Gherardi et al., 2015). Similarly, in Japan, of 75 *S. pyogenes* strains typed to *emm*1, 21.3% contained *mef* (A) gene (Wajima et al., 2008). However, this is not shown in current research, in which only 6(4.3%) *S. pyogenes* isolates harboring *erm* (A), with one strain containing *emm*1, explained probably by geographic variation. In addition, *tet* (M) was dominant resistance gene of tetracycline, comparable with our previous report of SDSE and GBS, demonstrating that *tet* (M) constituted the resistance mechanism of β-hemolytic *Streptococci* in China (Binghuai Lu et al., 2016; Lu et al., 2016).

The lower levofloxacin-resistant rate was reported in current study, making fluoroquinolone an alternative to β-lactams antibiotics in infections due to *S. pyogenes*. The development of fluoroquinolone resistance in *S. pyogenes* is mainly mediated by point mutations in the genes encoding DNA gyrase and topoisomerase IV subunit. High-level fluoroquinolone-resistant *S. pyogenes* strains are infrequently isolated, and there have been only limited reports from the United States (Biedenbach et al., 2006), Germany (Reinert et al., 2004), Taiwan (Lin et al., 2015), Portugal (Friães et al., 2013), and Japan (Wajima et al., 2013), distributed in *emm*11, *emm*12, *emm*28, *emm*58, and *emm*89. Comparatively, the nonsusceptible rate to fluoroquinolones (3.6%) in mainland China was far lower than that in Taiwan (11.1%), where the alterations of S79F and A121V in *parC* in *emm*12/ST36 strains were the most frequently mutations (Lin et al., 2015). In current study, there are 3 high-level resistant isolates (MIC > 256 μg/mL) and 2 immediate isolates (MIC = 3 mg/L), comparable to previous studies in China with 3.4 and 0% of them being fluoroquinolone-resistant (Liang et al., 2012; Yang et al., 2013). As previously documented, the mutations usually occur first in *parC*, conferring low-level fluoroquinolone resistance, then followed by mutations in *gyrA* that result in high-level fluoroquinolone resistance (Friães et al., 2013; Lin et al., 2015). Nevertheless, in current study, concurrent mutations in *parC* and *gyrA* did not exist always in high-level fluoroquinolone-resistant isolates. In 3 high-level resistant isolates distributed in various *emm* types, the resistance was caused by mutations in *parC* (A121V), *gyrA* (V192I), and both *gyrA* (S81F) and *parC* (S79F), respectively. Meanwhile, the significant relationship between *parC* mutation and *emm*/ST types in China was observed. All *emm*12 strains, resistant to levofloxacin or not, contained A121V.

Furthermore, our study is limited by a few factors. The sample size is relatively small, recovered only from 10 tertiary general hospitals of 7 cities/provinces in China. Moreover, the temporal and spatial distribution of the *S. pyogenes* isolates under study was unbalanced. Therefore, continuous studies with larger sample size are still in need to achieve comprehensive epidemic characteristics of *S. pyogenes* in China.

In summary, the *S. pyogenes* strains in present study showed some regional characteristics. The high resistance rate to erythromycin and clindamycin, a relatively high frequency of *emm* 12 and 1, close relationship between the point mutation of A121V in *parC* and *emm*12 strains, and high prevalence of *erm* (B) constitute significant characteristics of our *S. pyogenes* strains. The current study will contribute to the understanding of molecular characteristics of *S. pyogenes* circulating in mainland China and the decision making in the context of the M-protein-based vaccine developments.

## Author contributions

YaF, XC, JW, JZ, YL, DL, GZ, LH, HL, FZ, and YC isolated bacteria and performed the laboratory measurements. BL and DW made substantial contributions to conception and design. BL, YuF and DW wrote and revised the manuscript. BL drafted the manuscript. All authors read and approved the final manuscript.

### Conflict of interest statement

The authors declare that the research was conducted in the absence of any commercial or financial relationships that could be construed as a potential conflict of interest.
